# Immune Modulation by Transplanted Calcium Phosphate Biomaterials and Human Mesenchymal Stromal Cells in Bone Regeneration

**DOI:** 10.3389/fimmu.2019.00663

**Published:** 2019-04-02

**Authors:** Paul Humbert, Meadhbh Á. Brennan, Noel Davison, Philippe Rosset, Valérie Trichet, Frédéric Blanchard, Pierre Layrolle

**Affiliations:** ^1^Laboratory Phy-Os, Inserm UMR1238, University of Nantes, Nantes, France; ^2^Harvard School of Engineering and Applied Sciences, Harvard University, Cambridge, MA, United States; ^3^MERLN Institute for Technology-Inspired Regenerative Medicine, Maastricht University, Maastricht, Netherlands; ^4^Instructure Labs, B.V., The Hague, Netherlands; ^5^Centre Hospitalier Universitaire de Tours, Tours, France

**Keywords:** osteoimmunology, mesenchymal stromal cell, calcium phosphate biomaterial, bone regeneration, osteoclast, immune modulation

## Abstract

A wide variety of biomaterials have been developed as both stabilizing structures for the injured bone and inducers of bone neoformation. They differ in chemical composition, shape, porosity, and mechanical properties. The most extensively employed and studied subset of bioceramics are calcium phosphate materials (CaPs). These materials, when transplanted alongside mesenchymal stem cells (MSCs), lead to ectopic (intramuscular and subcutaneous) and orthotopic bone formation in preclinical studies, and effective fracture healing in clinical trials. Human MSC transplantation in pre-clinical and clinical trials reveals very low engraftment in spite of successful clinical outcomes and their therapeutic actions are thought to be primarily through paracrine mechanisms. The beneficial role of transplanted MSC could rely on their strong immunomodulatory effect since, even without long-term engraftment, they have the ability to alter both the innate and adaptive immune response which is critical to facilitate new bone formation. This study presents the current knowledge of the immune response to the implantation of CaP biomaterials alone or in combination with MSC. In particular the central role of monocyte-derived cells, both macrophages and osteoclasts, in MSC-CaP mediated bone formation is emphasized. Biomaterial properties, such as macroporosity and surface microstructure, dictate the host response, and the ultimate bone healing cascade. Understanding intercellular communications throughout the inflammation, its resolution and the bone regeneration phase, is crucial to improve the current therapeutic strategies or develop new approaches.

## Introduction

Bone regeneration strategies remain a critical challenge in the treatment of delayed union and non-union fractures (1), bone loss due to tumor resection (2), metabolic bone diseases, or to heritable skeletal dysplasia such as *osteogenesis imperfecta*. Autologous bone grafting is the current clinical gold standard to repair large bone defects. This entails harvesting the patient's own bone fragments, and transplanting them to the site of injury (3). There are ~2.2 million bone graft procedures performed annually worldwide, including 1 million procedures in Europe (4). Indeed, after blood, bone is the most frequently transplanted tissue. The significant disadvantages of bone grafting, including the severe pain and morbidity endured by patients as a consequence of the bone harvest site, have prompted advances in the development of synthetic biomaterials targeting bone repair. Human bone comprises ~70% of calcium phosphate (CaP) mineral; therefore CaPs are the biomaterials of choice to heal injured bone. They were first introduced in the 1920s as materials to facilitate bone repair (5) and have since undergone intense chemical and physical developments aimed at optimizing porosity, surface architecture, resorption rates, and mechanical strength in order to improve their bone healing capacities. Despite these advances in biomaterial design, CaPs still lack adequate osteogenecity to heal large, critical sized bone defects, and thus cell therapy has been employed for bone defect treatment with biomaterial bone substitutes such as CaPs to increase bone regeneration efficiency. Mesenchymal stromal stem cells (MSCs), derived primarily from the bone marrow and isolated by adherence to plastic, show great capacity for bone healing in unison with CaPs (6, 7). Although it is yet to be adopted into standard clinical practice, this state-of-the-art cell therapy is currently the most promising regenerative medicine strategy and has demonstrated successful bone healing in patients in clinical trials (8). The initial premise that MSCs, through cellular differentiation, regenerated damaged tissue was largely disregarded following observations that very few transplanted cells survive and engraft (9–11). Few children with severe *osteogenesis imperfecta* have received allogenic bone marrow transplant or allogenic MSC and showed faster growth, higher bone mineral content and less bone fracture than before transplant (12–16). Such growth and mineralization improvements were associated with <5% of donor cell engraftment. Consequently, it is proposed that the therapeutic benefit of transplanted MSCs is largely through a paracrine mechanism that stimulates recruitment of host cells, which ultimately form the new bone tissue. The underlying mechanisms involved have yet to be delineated, however evidence to date reveals that roles of MSCs and their secretions such as modulating immune responses (17), attenuating inflammation, and promoting angiogenesis (18), together act to ultimately ameliorate healing and restore function. The host immune-modulatory response to both CaPs and MSCs, encompassing both innate and adaptive immunity, and how this contributes to bone healing in the context of tissue engineered implants is the focus of the current review.

## Osteoimmunology of Calcium Phosphate Ceramics in Bone Regeneration

A wide variety of CaP biomaterials have been developed to fill bone defects as alternatives to autologous bone grafting. Synthetically synthesized ceramics mainly comprise sintered CaPs in order to achieve higher mechanical strength, including β-tricalcium phosphate (β -TCP), hydroxyapatite (HA), or their mixtures (biphasic calcium phosphate: BCP). These CaPs are therefore widely described in terms of their interactions with cells and tissues following implantation, as well as in relation to their bone forming abilities. Synthetic CaPs bioceramics are used successfully to fill bone defects in various clinical indications since they are considered biocompatible, bioactive and osteoconductive, thereby permitting guidance of the bone healing process (19). *In vivo*, the chemical and physical properties of the biomaterial dictate the host response and the ultimate bone healing cascade and osteoinduction has been achieved by various CaP ceramics, which demonstrate ectopic bone formation when implanted in the muscles or subcutaneously in animals [reviewed in (13)].While the interactions of these CaP materials with body fluids, cells, and tissues have been investigated at both the microscopic and ultrastructural levels, there is still a lack of understanding of the potential mechanisms leading to osteoinduction. Early on, the dissolution and precipitation of an apatite layer on CaP materials was identified as a potential major trigger for bone formation (20). It was further proposed that concentration of bone growth factors from body fluids, especially BMPs onto the biomaterial surface, attracts circulating stem cells to form bone tissue (21). The geometry of the biomaterial is certainly a critical parameter for bone induction. Studies demonstrate that in order for CaPs to exhibit osteoinductive properties, both a macroporous structure and surface microporosity are prerequisites. Micro- and macro- porous BCP biomaterials demonstrated the ability to induce mature lamellar bone tissue after 6 months without the addition of osteogenic cells or bone growth factors when implanted ectopically in sheep (22). Macro pores are introduced into CaPs by the addition of pore makers during the fabrication process. The importance of macrostructure in efficient osteoinduction is highlighted as bone formation occurs primarily in concavities (23). Microporosity is controlled by the sintering temperature, with lower sintering temperatures resulting in higher surface microporosity. Interestingly, the microporous CaPs bioceramics exhibited higher bone growth in critical size bone defects in goats compared with autologous bone grafts or the same CaPs bearing larger surface micropores and lower specific surface area (higher sintering temperature) (24). Increasing the microporosity increases the surface area thus possibly enhancing the dissolution/reprecipitation phenomenon (21). Further to biomaterial geometry, it has been speculated that low oxygen tension in the central region of the implants might provoke dedifferentiation of pericytes from blood vessels into osteoblasts (25). Most recently, Bohner and Miron added the idea that depletion of calcium and/or phosphate ions in the center of an implanted material could induce bone formation *via* the calcium-sensing of immune and bone cells (26).

In early reports, bone induction by CaPs ceramics was thought to be limited to the muscles of large animals such as rabbits, sheep, goats, dogs, and baboon, until Barradas et al. screened various different mouse strains and found osteoinduction by CaPs ceramics in FVB/NCrl mice (27). This study was a major step for further understanding the biological mechanisms of osteoinduction by these ceramics because there are abundant immunohistochemistry protocols available for mice compared to large animals, not to mention their ease of handling and low cost.

### Innate Immune Response to Calcium Phosphate Biomaterials

Various innate immune cells participate in the host-cell response to the implantation of CaP materials including mast cells, neutrophils, monocytes, macrophages, and multinucleated giant cells (MNGCs) (28). In addition to their role in the innate immune response, macrophages have tissue-specific functions. Osteal macrophages (so called OsteoMacs), a specific type of specialized macrophages residing in the periosteum and endosteum, are an important cell type for the regulation of bone healing (29) but less is known about their relationship with implanted biomaterials (30). Depletion of OsteoMacs in mice demonstrates their key role in regulating bone regeneration in normal bone healing in a bone injury model (31, 32), suggesting that resident macrophages may also possess the phenotypic capability to instruct bone regeneration upon implantation of biomaterials used for bone repair. Previous studies have documented that resident or infiltrating monocyte-derived macrophages present at early time points after tissue trauma or the implantation of a biomaterial are characterized as pro-inflammatory (M1 macrophages), typified by their secretion of inflammatory cytokines such as TNFα, IL-1, IL-6, and IL-12, while macrophages present at later time points exhibit a predominantly anti-inflammatory profile (M2 subtype) and promote healing by secretion of cytokines such as IL-10 and TGF- β, stimulating angiogenesis, and recruiting cells for tissue repair (33–36). Importantly, macrophage polarization can be switched between M1 and M2, rendering them highly sensitive and adaptive to their environment. Moreover, mounting evidence suggests that macrophage polarization occurs over a continuous spectrum, rendering the M1/M2 classification paradigm too simple to accurately characterize their dynamic phenotypic changes and plasticity *in vivo*. In any case, macrophages are among the first cells present at the site of CaP implantation and play an integral role in MSC migration and bone formation (Table 1). The infiltration of macrophages and the subsequent homing of MSCs and ectopic bone formation was observed after CaP implantation in mice (44). Interestingly, MSCs migration and osteogenic differentiation was significantly enhanced by conditioned media (CM) from macrophages cultured on BCP, compared to CM from macrophages cultured on tissue culture plastic (43, 44). Furthermore, it was shown that macrophage-secreted MCP-1 and MIP-1α were the effectors of enhanced MSC migration.

**Table 1:** Implication of macrophages and osteoclasts in the bone formation induced by calcium phosphate biomaterials.

**CaP biomaterial**	***In vitro* and *in vivo* models**	**Outcome**	**References**
Hydroxyapatite (HA)	*In vitro:* Osteoclasts (OCs) were differentiated from bone marrow monocytes from C57BL/6 mice. Primary osteoblasts (OBs) were derived from the calvaria. *Ex vivo:* Organ culture of explanted calvaria. *In vivo model:* C57BL/6mice	CTHRC1 protein is secreted by mature OCs. CTHRC1 mRNA expression is elevated in OCs cultured on HA compared to tissue culture plastic (TCP). CTHRC1 stimulates osteoblastogenesis (gene expression and mineralized matrix deposition). CTHRC1 expression and bone turnover *in vivo* was increased by RANKL injections and conversely decreased by alendronate treatment. OC-specific CTHRC1 KO mice led to reduced bone formation and lower bone mass.	(37)
Coral derived calcium carbonate (CC)/ HA constructs	*In vivo model:* Intramuscular implantation in Chacma baboons	Osteoinduction of biomaterials was inhibited by preloading constructs with the bisphosphonate zoledronate.	(38)
β-TCP	*In vivo model:* Intramuscular implantation in female beagle dogs	CaP induces the formation of TRAP and Cathepsin K positive, multinucleated cells on the biomaterial, and their presence precedes ectopic bone formation	(39)
β-TCP with different surface microstructures	*In vitro:* Osteoclasts were differentiated from a murine macrophage cell line RAW264.7 Human MSCs were isolated from bone marrow harvested from femoral heads. *In vivo model:* Intramuscular implantation in male mongrel dogs	*In vitro*, CaPs with submicron-scale surfaces lead to increased differentiation of OCs and higher secretions of factors that induced osteogenic differentiation of MSCs. *In vivo*, submicro-structured CaPs formed bone and OCs presence was significant, whereas micro-structured CaPs formed no bone and OC presence was spare.	(40)
β-TCP	*In vivo model:* Rabbit femoral condyles	Loading of Alendronate (bisphosphonate) onto β-TCP inhibited the presence of TRAP-positive cells on the surface of the biomaterial and abrogated the CaP-mediated bone formation.	(41)
β-TCP	*In vivo model:* FVB/NCrl strain mice	CaPs induced osteoclastogenesis and ectopic bone formation. Depletion of osteoclasts by local injection of liposome-encapsulated clodronate impeded bone formation by CaPs.	(42)
Biphasic calcium phosphate (BCP) HA/ β-TCP composite	*In vitro:* Mouse macrophage cell line RAW264.7. Mouse bone marrow-derived MSCs.	Macrophages upregulated gene expression of inflammatory factors (IL-1, IL-6, MCP-1) and growth factors (EGF, PDGF, and VEGF) as a consequence of their CaP substrate. This macrophage conditioned media (CM) increased MSC migration and osteogenic differentiation (osteogenic gene expression and mineralized matrix deposition).	(43)
BCP (HA/ β-TCP)	*In vitro:* Mouse macrophage cell line RAW264.7. Mouse bone marrow-derived MSCs. *In vivo model:* Implantation into thigh muscle of male BALB/c mice.	BCP implantation *in vivo* caused infiltration of macrophages to the site, followed by homing of MSCs and subsequent ectopic bone formation. BMSCs migrated significantly faster under stimulation by CM from macrophages cultured on BCP, compared to CM from macrophages cultured on TCP. Secretion of MCP-1 and MIP- 1α by macrophages was increased by culture on BCP and were shown to be the effectors of enhanced migration since blocking these in macrophage CM had inhibited MSC migration.	(44)

Osteoclasts, which originate from the same hematopoietic precursor as macrophages, are multi-nucleated cells capable of efficiently degrading both the organic and inorganic fractions of bone. Activated osteoclasts have a characteristic morphology including a ruffle border by which they secrete proteases, such as cathepsin K and matrix metalloproteinases, and release hydrogen ions by proton pumps to acidify the resorptive pit. Histologically, osteoclasts can be identified by intensely positive tartrate-resistant acid phosphatase (TRAP) activity, which relates to their functional activity in resorbing bone or mineralized substrates such as CaPs (45). Osteoclastogenesis is essentially regulated, both *in vivo* and *in vitro*, by the macrophage colony-stimulating factor (M-CSF) and the tripartite system constituted by the receptor activator of nuclear factor κB (RANK), its ligand (RANKL) and osteoprotegerin (OPG). M-CSF permits survival and proliferation of osteoclast-precursors, also allowing them to respond efficiently to RANKL stimulation. RANKL triggers differentiation into osteoclasts by binding RANK, while OPG can prevent the interaction as a decoy receptor for RANKL (46). Osteoclasts are important players in the bone healing cascade. Several studies have documented that osteoclast presence at the site of CaP implantation precedes new bone formation (39). Evidence to demonstrate the crucial interplay between osteoclasts and osteoblasts, in association with CaPs, was highlighted by several studies (Table 1). Bisphosphonates are a class of drug employed to inhibit bone resorption by induced osteoclast apoptosis (47). The first-line medical management for *osteogenesis imperfecta* is based on bisphosphonates to inhibit osteoclasts, while the disease relies on osteoblast dysfunction. Bisphosphonates allow an increase of bone mineral density and a 20% decrease of fractures in long-bone in the pediatric *osteogenesis imperfecta* population (48, 49). However, in CaP-mediated bone formation, several osteoclast depletion strategies including the administration of bisphosphonates highlight the important role of osteoclasts, suggesting that coupling mechanisms linking osteoclast resorption to osteogenesis may be involved (50). Of note, Takeshita et al. convincingly showed that osteoclasts in association with CaP or bone secrete CTHRC1, which enhances osteoblastogenesis, thereby coupling bone resorption to formation. CTHRC1-triggered bone turnover was attenuated when resorption was inhibited by bisphosphonate (alendronate) treatment, and OC-specific CTHRC1 KO mice led to reduced bone formation and lower bone mass (37). This concurs with findings by other groups that bisphosphonates inhibited osteoclasts and osteoinduction by CaPs in baboons (38) or rabbits (41). Furthermore, depletion of osteoclasts by local injection of liposome-encapsulated clodronate impeded heterotopic bone formation by intrinsically osteoinductive microstructured CaPs after subcutaneous implantation in mice (42). Surface microstructure stimulates osteoclastogenesis and therefore may be a primary trigger for subsequent *de novo* bone formation for certain CaPs which do not require the addition of MSCs or growth factors to induce bone formation (40). The biological mechanism by which osteoclasts stimulate subsequent osteogenesis in response to these microstructured CaPs is still not understood. Even more interesting, non-microstructured CaPs, which possess no intrinsic osteoinduction potential, have been show to induce heterotopic bone formation when first seeded with osteoclasts prior to implantation. Taken together, OC depletion and enrichment strategies combined with implanted CaPs points to an essential role of this cell type in inducing new bone formation

Distinct from osteoclasts, MNGCs are observed in human histological samples around various CaP bone substitutes and their presence correlates with a higher maintenance of bone mass in grafted sites (51). Such MNGCs are formed by fusion of monocytes/macrophages on various bone substitutes not surrounded by bone. Histologically, they are slightly TRAP positive and occasionally associated with small resorption lacunae, indicating a potential osteoclast-like activity. *In vitro*, they can be obtained by stimulation of monocytes with IL-4 and IL-13 (52, 53). These *in vitro* generated MNGCs can dissolve hydroxyapatite, although not as efficiently as osteoclasts, but they cannot digest the bone matrix (54). The case *in vivo* may however be more complex, particularly since mononucleated and fused macrophages found at the surface of implanted biomaterials or wounds may express a variety of markers spanning both classical M1 and alternatively activated M2 phenotypes.

Dendritic cells (DC) have been described as the scavenging sentinel cells also responsible for identifying foreign materials and organisms in the host. Although 25% of monocytes present at the site of injury or inflammation differentiate into DCs, the current knowledge of how DCs interact with biomaterials is incomplete—particularly whether they interact with the foreign body distinctly or in concert with macrophages and MNGCs (55). This is compounded by the heterogeneity of DC subsets, similar to macrophages (56). Still, it is clear that DCs also possess phagocytic ability and can readily internalize CaP particles or polymeric beads. Such particle internalization causes DCs to secrete inflammatory cytokines as well as migrate back to the lymph nodes and instruct the adaptive immune response through T cell priming (55, 57). Because these cells interrogate and recognize foreign bodies as well as prolifically express surface antigens, DCs represent an important bridge between the innate and adaptive immune system and may mediate the polarization or transition between inflammatory or anti-inflammatory adaptive immunity. Illustrating this immune-modulatory role, DCs have been implicated with suppression of a chronic inflammatory response to implanted biomaterials and thus may play a key role in mediating the transition from fibrous encapsulation to functional tissue regeneration, and as the case may be with CaPs implanted in bony locations, the regeneration of bone tissue. Similar to macrophages, DCs have been shown to distinctly respond to biomaterial surface chemistry, hydrophobicity, and topography which direct activated vs. suppressive states of DCs (58). Some work has been conducted to explore the role of DCs in mediating the innate and adaptive immune response to subcutaneously implanted polymeric materials *in vivo* (59), but less is known about how DCs may interact with resorbable biomaterials such as calcium phosphates, particularly those that are too large to phagocytose.

These studies emphasize the crucial role of the innate immune system and osteoclastogenesis in modulating and facilitating bone healing and how CaP biomaterial properties such as surface microporosity significantly affect such responses. It should be noted that the combination of CaP biomaterial and natural (collagen, fibrinogen etc.) or synthetic polymers are also developed to influence the osteoinductive capacities of the implant (60) and could therefore influence the immune response. In spite of the significant improvements in CaPs, yielding well tolerated, osteoconductive biomaterials with some osteoinductive capability, most CaPs still lack adequate osteoinduction capacity for regenerating large bone defects. Therefore, they are generally employed for treating small bone defects, to supplement autologous bone grafting, or, increasingly, as scaffolds to deliver cells or growth factors targeting bone repair (61, 62).

## Osteoimmunomodulation and Osteoinduction by MSC/CaP Combinations

Bone marrow derived mesenchymal stromal cells may overcome the challenges of autologous bone grafting for the regeneration of large defects. Transplanted in unison with CaP bioceramics, MSCs achieve ectopic (intramuscular and subcutaneous) (7, 9, 63) and orthotopic bone formation and critical-sized defect healing in preclinical studies, and efficient fracture healing and bone augmentation in clinical trials (64, 65). The key role of implanted MSCs was initially thought to be their differentiation into bone forming osteoblast cells and studies observing transplanted MSCs within osteocyte lacunae of newly formed bone support this hypothesis (6, 66–68). However, in general, cell engraftment of transplanted MSCs is very low or completely absent, in spite of successful outcomes (10, 11, 69), leading to the contention that the therapeutic benefit of transplanted MSCs is largely through a paracrine mechanism. These conflicting observations of the fate of transplanted MSCs is present throughout the literature and could be caused by a multitude of reasons such as initial cell dosage, biomaterial scaffold employed, implantation site, and host immune response. In our own hands, we have observed instances of some, albeit a small proportion, transplanted MSCs present in newly formed bone (9), and others where cell engraftment was not detected (10), while both resulted in ectopic bone formation. Although not quantified, it appears the transplanted MSCs persisted in outcomes of abundant bone formation and interestingly human MSCs resided in osteocyte lacunae in the vicinity of host (mice) osteocytes, with host osteocytes representing the larger proportion (9). MSCs secrete a vast array of paracrine factors into their conditioned media (MSC-CM) *in vitro* and interestingly, administration of MSC-CM *in vivo*, induces healing in many tissues including bone (70–72) providing evidence that the MSC secretome can initiate the bone tissue regeneration cascade. The MSC secretome comprises all factors secreted by MSCs, including soluble secretions (cytokines, growth factors, chemokines, and hormones) as well as vesicular secretions, or extracellular vesicles (EVs), which encompass exosomes, microvesicles, and apoptotic bodies. EVs are nanoparticles (ranging in size from 30 to 1,000 nm) that are secreted by all cells and carry bioactive cargo from the parental cells including lipids, proteins, RNA, and DNA (73, 74). It was recently reported that EVs secreted by MSCs have therapeutic potential in preclinical studies targeting bone repair (75–78). While not yet investigated in the context of bone regeneration, it has been observed in other settings that EVs secreted from MSCs mimic the immune-regulatory function of MSCs (79).

### The Immune System Influences MSC-Based Bone Regeneration

Several studies have observed that MSCs enhance bone repair by modulating the foreign body response to CaPs. Macrophages are an important innate immune cell population for the regulation of MSC-based bone regeneration. Interestingly, it was observed that the mobilization of macrophages to the site of CaP implantation was significantly enhanced by MSC transplantation prior to MSC-mediated ectopic bone formation (10, 17). Early studies indicated that inflammatory macrophages suppressed osteoblastogenesis, through secretion of TNFα and IL1b [reviewed in (50)]. However, in contrast to this, both Tour et al. (17) and Gamblin et al. (10) independently observed that transplanted MSCs led to a M1 dominant macrophage phenotype, which was followed by bone formation. In line with these *in vivo* studies, several *in vitro* studies have demonstrated the impact of M1 macrophages on enhancing the osteogenic differentiation of MSCs. We previously demonstrated that inflammatory M1 macrophages secrete Oncostatin M (OSM) to improve osteoblastogenesis *in vitro* (80). In addition, OSM production by macrophages sustained bone regeneration in a mouse model of tibia injury (81). Furthermore, MSCs treated with conditioned media (CM) from lipopolysaccharide (LPS) stimulated monocytes exhibited increased osteogenic differentiation (82), an effect partially imparted by extracellular vesicles secreted by the activated monocytes (83). Conversely, other *in vitro* studies have reported that M2, and not M1 macrophages, enhanced osteogenic differentiation of MSCs (84). The exact role of resident vs. monocyte-derived macrophages or of M1 vs. M2 alternatively activated macrophages in response to transplanted MSCs are still not clear. The M1/M2 paradigm is certainly a key for successful bone regeneration, since resolution of inflammation and tissue repair are tightly linked (85). Interestingly, M1 and M2 macrophages were both recently demonstrated to modulate MSC osteogenic differentiation but in disparate manners, whereby M1 macrophages enhanced early osteogenic differentiation without any effect on matrix mineralization, which was subsequently enhanced by M2 macrophages (86). In addition, it was demonstrated that macrophages preferentially recruit fibroblasts over MSCs. Pre-incubation of macrophages with immunomodulatory MSCs impairs fibroblast recruitment (87). Taken together, these studies indicate that macrophage polarization is important for distinct roles in the bone healing cascade by MSCs in association with CaPs, much like how normal tissue repair encompasses a transition from a pro-inflammatory status to a pro-reparative status.

Osteoclasts also play a central role in the regulation of MSC-based bone regeneration. It was demonstrated *in vitro* that osteoclasts secrete factors (S1P, BMPs, WNTs etc.) which induce MSC migration and osteogenic differentiation (88, 89). Interestingly, MSCs transplanted with BCP were shown to positively influence the foreign body reaction by attracting circulating monocytes and inducing their differentiation into osteoclasts, thus favoring bone formation. Importantly, depletion of osteoclasts by local injection of clodronate or injection of neutralizing anti-RANKL antibodies impeded bone formation, highlighting the imperative role of osteoclasts in MSC-mediated bone formation (10).

The adaptive immune system also plays an important role in MSC-modulated bone regeneration, which was elegantly shown by Liu et al. (90) and is discussed in detail in Table 2. Briefly, MSCs together with CaP particles induced ectopic bone formation in immuno-deficient mice but failed to do so in immune competent C57BL/6 mice (90). Moreover, infusion of CD4+ T cells in nude mice blocked ectopic bone formation through secretion of TNFα and IFNγ, which inhibited MSC differentiation and induced MSC apoptosis (90, 92). Interestingly, infusion of CD4+ CD25+ Treg abolished TNFα and IFNγ production and improved MSC-mediated bone regeneration in critical-sized calvarial bone defects in C57BL/6 mice (90). These observations were corroborated by findings that MSC from immune-competent mice formed ectopic bone in immune deficient mice, but much less in syngenic mice with the initiation of an inflammatory reaction involving Th1, Th2, and cytotoxic T-cell responses (91). Collectively these data demonstrate that modulation of both the innate and adaptive host immune response facilitates MSC-based bone regeneration.

**Table 2:** Osteoimmunology of mesenchymal stem cells transplantation with calcium phosphate biomaterials.

**CaP Biomaterial**	**MSC origin**	***In vitro* and *In vivo* models**	**Outcome**	**References**
BCP (HA/ β-TCP)	Human bone marrow derived MSCs	*In vivo model:* Intramuscular implantation in immunocompromised nude NMRI Nu/Nu female mice	Both macrophage and osteoclast presence at the CaP site was significantly enhanced by MSC transplantation. Their presence preceded MSC-mediated ectopic bone formation. Depletion of osteoclasts by local injection of clodronate impeded bone formation, highlighting the imperative role of osteoclasts in MSC-mediated bone formation	(10)
HA	Rat (Lewis) bone marrow derived MSCs	*In vivo model:* Rat calvaria critical-sized defects	MSCs increase bone formation by modulating (both up- and down-regulation) the foreign body reaction. MSCs increased macrophage presence at the CaP implantation site and enhanced bone healing. However, MSCs reduced the immune cell presence (macrophages and eosinophils at the site when the scaffold was delivered with extracellular matrix produced by fibroblasts (dermis of Sprague-Dawley rats), indicating that MSCs modulate the host immune response depending on the environment with the aim of positively influencing the tissue healing cascade.	(17)
BCP (HA/ β-TCP)	Bone marrow MSCs C57BL/6-Tg (CAG-EGFP)1Osb/J mice	*In vivo model:* Subcutaneous and calvaria implants. Female C3H/HeJ, C57BL6J, B6.129S7-Ifng^tm1Ts/^J, C57BL/6-Tg(CAG-EGFP)1Osb/J, B6.MRL-Fas^lpr^/J, immunocompromised nude mice (Beige nude/nudeXIDIII).	Firstly, MSC transplantation with CaP formed ectopic bone in nude mice but not in C57BL/6 mice. Interestingly CD8+ T cells, and CD4+ T cell infusion into nude mice partially and totally blocked bone formation, respectively. Inhibition of MSC-mediated bone formation in C57BL/6 was caused by interferon (IFN)-γ induced down-regulation of the runt-related transcription factor 2 (Runx-2) pathway and tumor necrosis factor (TNF)-α-induced MSC apoptosis. Treatment with IFN-γ and TNF-α also inhibited MSC-mediated bone formation in nude mice and interestingly antibodies to neutralize IFN-γ and TNF-α, as well as infusion of Treg cells rescued bone formation by transplanted MSCs in C57BL/6 mice. Together, this reveals that pro-inflammatory T cells inhibit transplanted MSC-mediated bone repair.	(90)
BCP (HA/ β-TCP)	Bone marrow MSCs from C57BL/6 mice	*In vivo model:* Subcutaneous implantation in C57BL/6 and immunocompromised nude mice (NMRI Nu/Nu)	MSC transplantation into nude mice led to abundant ectopic bone and bone marrow formation, whereas MSC transplantation into syngenic C57BL/6 mice resulted in only minor quantities of ectopic bone formation and significant quantities of multinucleated giant cells (MNGCs). MSCs survived for a shorter duration in immune-competent mice and the implant site was characterized by Th1, Th2, and cytotoxic T-lymphocyte activation, highlighting the benefit T-lymphocyte absence in nude mice for bone formation.	(91)

## Impact of MSC Stress on Immunomodulation

As indicated above, implantation of MSCs with CaP results in the local recruitment of various innate immune cells including mast cells, neutrophils, monocytes, macrophages, and several types of multinucleated giant cells. An exhaustive overview of how MSC influence the innate and adaptive immune system is outside the scope of this review. Rather, we focus on how transplanted MSCs in association with CaPs may modulate the immune system by focusing on the conditions that MSCs encounter following transplantation and the potential impact that these cell stresses can have on MSCs immunomodulation.

### MSC Influence the Innate and Adaptive Immune System

**Figure 1:**
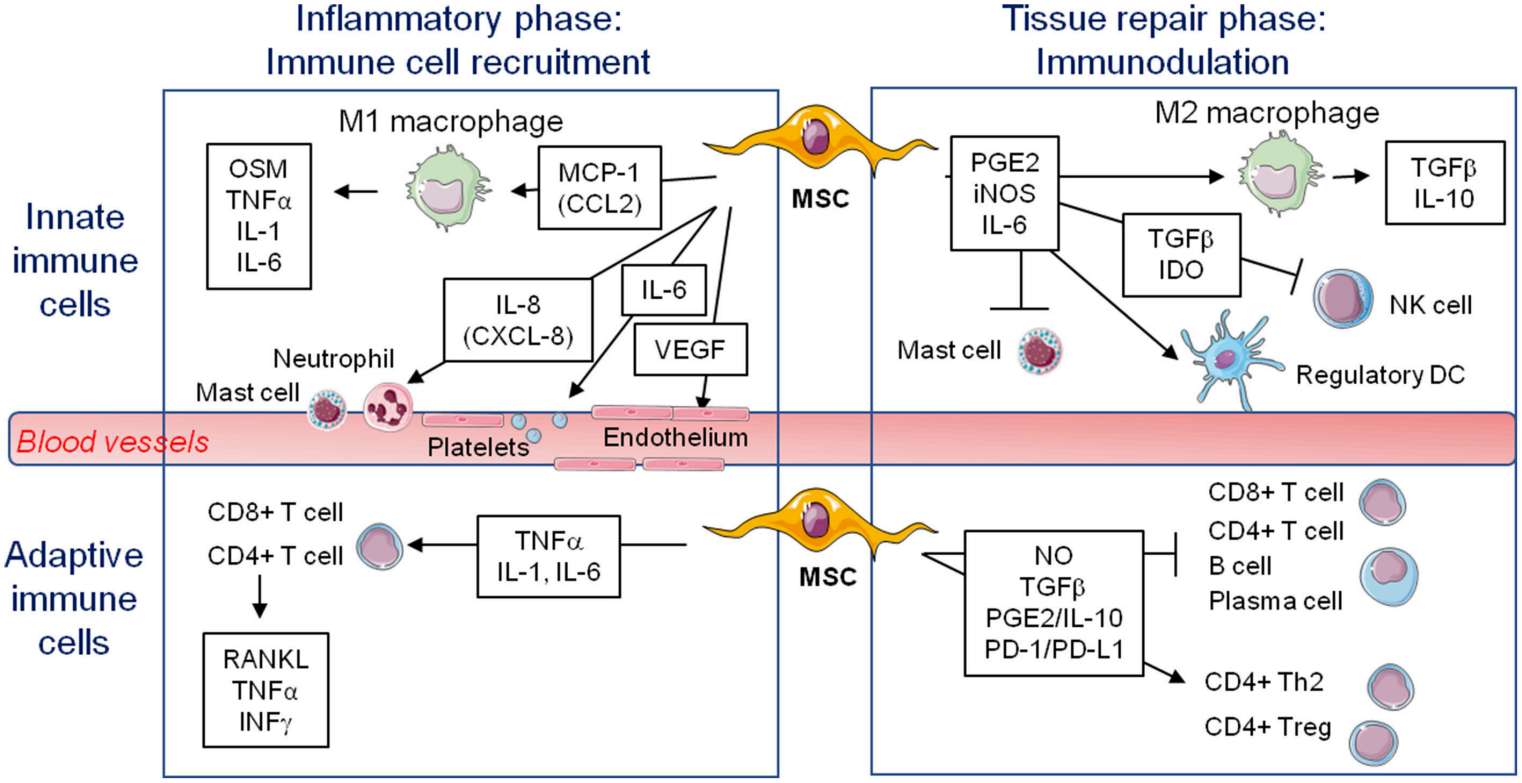
Known immunomodulatory secretions from mesenchymal stem cells favoring (↑) or inhibiting (-) various cells potentially involved in bone formation on a biomaterial during early inflammation or the later stage of tissue repair. MSC, mesenchymal stem cell; M1, pro-inflammatory macrophages; M2, alternatively activated macrophages; NK, natural killer; DC, dendritic cell; OSM, oncostatin M; TNFα, tumor necrosis factor alpha; IL, interleukin; VEGF, vascular endothelial growth factor; PGE2, prostaglandin E2; iNOS, inducible nitric oxide synthase; TGFβ, transforming growth factor beta; IDO, indoleamine 2,3-dioxygenase; RANKL, receptor activator of nuclear factor kappa-B ligand; IFNγ, interferon gamma; NO, nitric oxide; PD-1/PD-L1, programmed cell death protein 1/programmed cell death-ligand 1.

Since MSCs express low levels of MHC-II and costimulatory molecules (CD40, CD80, CD86), but substantial amount of the tolerogenic HLA-G molecule, they are considered as immunoprivileged cells, and thus would be ideal for tissue repair even in allogeneic transplantation (92, 93). Moreover, the discovery of the immunomodulatory roles of MSCs fostered their therapeutic use to suppress inflammation and limit pathogenic immune responses in graft-vs-host and auto-immune diseases such as multiple sclerosis, diabetes, and rheumatoid arthritis. Indeed, MSCs tend to limit macrophage polarization to M1, favoring M2 polarization. They also favor the generation of regulatory dendritic cells. They inhibit mast cells degranulation and NK cell effector functions (Figure 1). MSC production of PGE2, IL-6, TGFβ, and IDO for example has a key role in these suppressive effects on innate immune cells (93, 94). With regard to adaptive immune cells, MSCs favor the development of Th2 and Treg cells, with suppression of CD4+ T cells proliferation and polarization toward Th1 and Th17 cells. They also inhibit B cell activation, proliferation, and differentiation into plasma cells. These suppressive effects depend on MSCs production of NO, TGFβ, PGE2, IL-10, and ligation of PD-1/PD-L1 for example (93, 94). Interestingly, culture of MSC on BCP did not impair their suppressive effect toward T, B, and Natural Killer (NK) cells (95). Extracellular vesicles produced by MSC are also implicated in immunomodulation (96). It is important to note that the immunosuppressive effect of MSCs when delivered systemically is well documented, but the possible role of MSCs in regulating the innate and adaptive immune responses when delivered locally to regenerate bone remains elusive.

### Impact of Stressful Conditions on MSCs Phenotype/Secretome

Because MSCs disappear shortly after implantation with CaP, it is important to consider the impact of cell stress or cell death on MSCs immunomodulation activity. The primary factors responsible for the large cell death of transplanted BMSCs include the ischemic environment and the lack of glucose that the BMSCs encounter (97–100). It is unclear the exact means of MSCs death after implantation with CaP but senescence, apoptosis, necrosis, or other types of cell death could presumably be implicated which can have a profound effect on MSC-mediated immunomodulation. MSCs are considered relatively resistant to programmed apoptosis and prefer senescent growth arrest or autophagy to cell death (101). In general, necrotic (necroptotic, pyroptotic) cell death is associated with inflammation and exacerbated immune responses, whereas apoptosis avoids an inflammatory response and rather contributes to its resolution. For example, Laing et al. demonstrated that systemic injection of H_2_O_2_-induced apoptotic MSCs is more efficient than injection of live MSCs to induce a robust immune suppressive reaction in an ovalbumin induced model of allergic airway inflammation (102). Similarly, Galleu et al. showed that after infusion of apoptotic MSCs in a murine model of graft-vs-host disease, recipient phagocytes engulf apoptotic MSCs and produce IDO, which is ultimately necessary for effecting immunosuppression (103). The authors also observed that cytotoxic cells, such as CD8+ T lymphocytes and NK cells, induce MSCs apoptosis through perforin, granzyme B, and FasL, and that PBMCs from patients that responded to MSC therapy had more cytotoxic activity against MSCs. Another level of complexity is that when apoptotic cells are not cleared in an efficient and timely manner, they progress to secondary necrosis and lose their membrane integrity. This results in a leakage of immunostimulatory, danger associated molecular patterns (DAMPs) such as HMGB1 and nucleosomes (104, 105). They induce an inflammatory response which can become chronic and even induce an adaptive immune response, a situation that would presumably preclude local bone formation. Additional studies are mandatory in the context of bone regeneration induced by MSC-CaP combination.

Upon aging and in age-related deficiencies, compromised MSC-mediated immunological responses have been observed and attributed to MSC senescence. Senescence by replicative exhaustion or genotoxic stress during *ex vivo* culturing was also demonstrated (69). Acute, transient senescence induced by cell stresses such as hypoxia is presumably beneficial, because senescent cells secrete a plethora of molecules as part of the senescence-associated secretory phenotype (SASP), leading to rapid MSC clearance by immune cells, modulation of innate and adaptive immune cells, followed by tissue healing and regeneration (106). However, when chronic senescence occurs, for example upon aging, it impacts on the SASP, the local microenvironment and causes local and/or systemic inflammation.

The modifications of the secretome of MSCs induced by various stimuli, either mimicking physiological situations such as hypoxia and inflammatory stress or specific *in vitro* culture conditions to enhance the immunomodulatory properties of the cells, were previously widely reviewed (107–109). Those stresses could also alter the production and composition of EVs (110–112). Hypoxia is a main characteristic of the natural environment of MSCs and a major difference with *in vitro* culture. Overall, culture under low-oxygen atmosphere results in higher proliferation rate, survival, differentiation potential, and immune modulating secretions (113). For example, Paquet et al. (114) reported an upregulation of proangiogenic and chemotactic mediators (VEGF-A/-C, IL-8, MCP-1, and RANTES) and a downregulation of inflammatory mediators (IL-1b, IL-6, IL-15, IL-1Ra) with close to anoxic conditions (0.1% O_2_). An artificial overexpression of the hypoxia-inducible factor 1 (HIF-1) in dental stem cells leads to an improved resistance to NK cells, an upregulation of CXCL12, CCL5, and IL-6 as well as a downregulation of CXCL10 (115).

Inflammatory stress is also characteristic of an implantation site and is mimicked *in vitro* by exogenous addition of LPS, TNFα, and/or IFNγ, usually termed MSC priming. When primed with inflammatory cytokines, MSCs increase their suppressive capacities (95). MSCs express constitutively many mitogenic growth factors, chemokines and matrix metalloproteinases at various levels. They are sensors and modulators of their microenvironment; i.e., MSC response to TNFα by increasing expression of some growth factor receptors, growth factors, chemokines, and matrix metalloproteases (116). Just as hypoxia, MSCs stimulated with LPS or TNFα produced more VEGF and FGF2 but also more HGF and IGF-1 via the activation of NFκB (117). Stimulation with IFNγ increases the expression of anti-inflammatory and regenerative molecules such as IDO, TGFβ or PGE2 for example (60). The addition of hypoxia to a TNFα and IFNγ stimulation on adipose-derived stem cells did not impair their higher secretion of immunomodulatory molecules IDO and PD-L1 (118).

## Proposed Mechanism of Bone Formation After MSC-CaP Implantation

**Figure 2:**
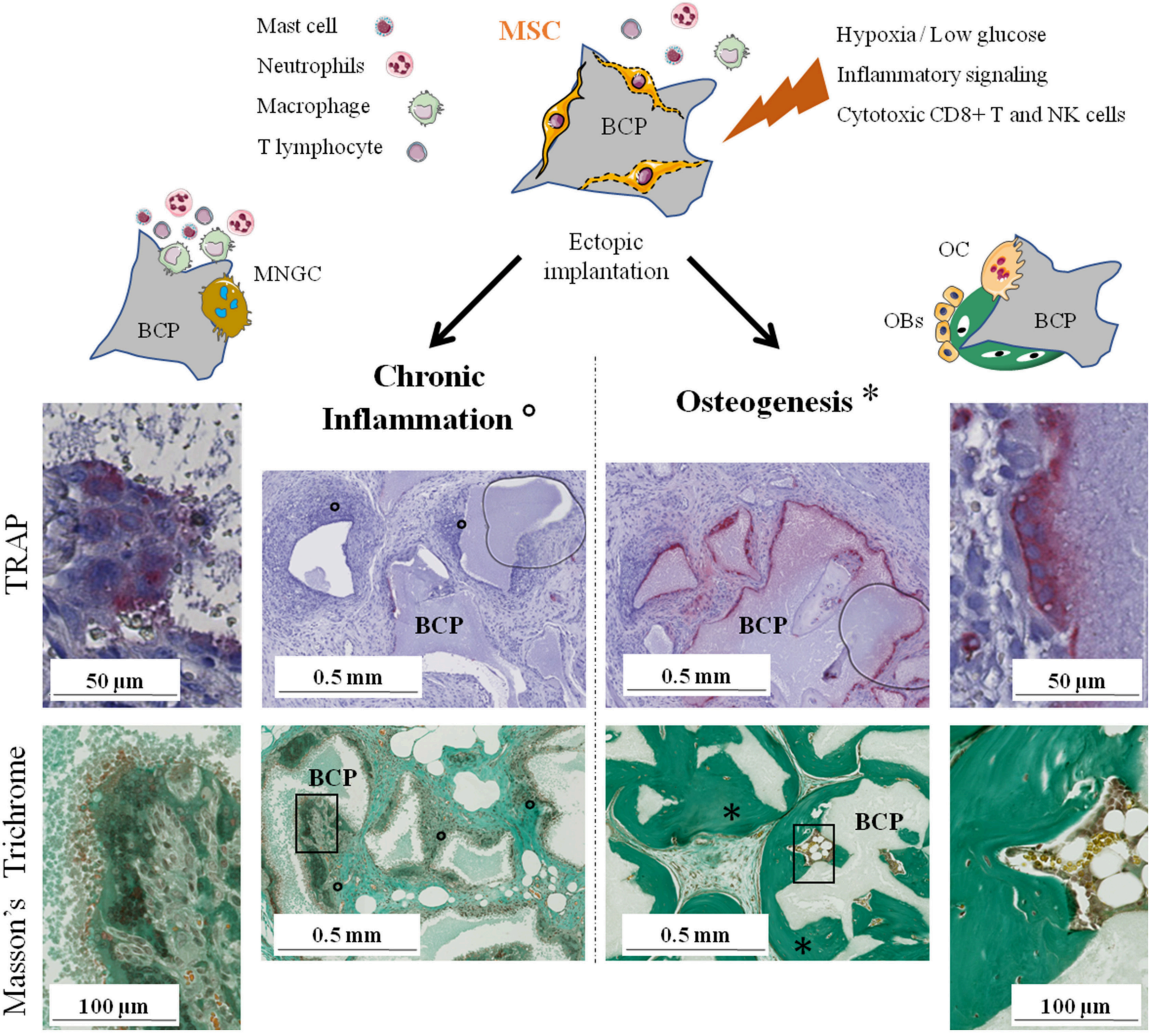
The two possible outcomes of subcutaneous implantation of mesenchymal stem cells on calcium phosphate ceramic in mice. Histology of the implants: TRAP staining for osteoclasts detection after 4 weeks and Masson's trichrome to evaluate bone formation after 8 weeks. On the left, chronic inflammation (o) with formation of TRAP negative MNGCs followed by fibrous encapsulation and no sign of bone formation. On the right, osteoclastogenesis on the biomaterial followed by abundant bone formation (^*^). NK, natural killer; BCP, biphasic calcium phosphate; MNGC, multi-nucleated giant cell; OC, osteoclast; OBs, osteoblasts; TRAP, tartrate-resistant acid phosphatase.

It has been shown in many studies that only the combination of CaP and MSCs has the ability to induce abundant bone formation. MSCs have numerous, complex, and sometimes antagonist effects on the immune system depending on the physiological context. Their role in bone regeneration on CaP biomaterials remains unclear but evidence indicate that their immunomodulatory properties are involved. We previously highlighted the crucial role that osteoclasts seem to play and the rapid disappearance of implanted MSCs before new bone is formed. Therefore, we hypothesize that MSCs, through their dialogue with various cells of the immune system, favor osteoclastogenesis on lieu of MNGCs formation, i.e., inducing a switch from chronic inflammation and fibrous encapsulation to bone formation via the recruitment and differentiation of new MSCs or skeletal stem cells in the bone remodeling process (Figure 2).

In detail, the environment just after implantation consists of the biomaterial exhibiting specific properties (chemical composition, micro-/macro-porosity, topography) and the MSCs adhering and reacting to it. Neutrophils, mast cells and macrophages are the first immune cells in contact with the implant, the latter mostly polarizing toward the inflammatory M1 phenotype (28). Therefore, inflammatory cytokines, ions released by the biomaterial, lack of O_2_ (98), and nutrients (97), presence of cytotoxic CD8+ T and NK cells are all environmental factors influencing MSCs' behavior in the early stages of implantation. Most of those stresses were individually found to increase the production of pro- or anti-inflammatory molecules by MSCs (107–109). Given the osteogenic effect of the biomaterial (119) and the M1 population of macrophages (86), implanted MSCs might also express some markers of early osteoblast precursors. Eventually, MSCs will disappear by senescence, apoptosis and/or necrosis, releasing novel pro- and anti-inflammatory signals. Clearance of dead MSC by immune cells would also modulate the innate and adaptive immune system.

We believe that the secretions from those highly stimulated MSCs directly or indirectly (through modulation of innate and adaptive immune cells) favor the formation of osteoclasts at the expense of MNGCs. Indeed, MSC-based bone formation was significantly altered by anti-RANKL mAB (10) or clodronate (42) administration. While clodronate also affects MNGCs, the anti-RANKL mAB is specifically restricting osteoclastogenesis. Due to their common origin and similar morphology, osteoclasts, and MNGCs are difficult to distinguish. Theoretically, both osteoclasts and MNGCs can arise from the fusion of circulating monocytes, M1/M2 macrophages or even of dendritic cells. An in depth description of the known differences between osteoclasts and MNGCs have already been well reviewed (120). Both cell types share a lot of markers but they can be differentiated by expression of the calcitonin receptor and RANK only in osteoclasts, or CD86 (B7-2), CD206, and HLA-DR only present in MNGCs. Interestingly, MNGCs are able to express low levels of TRAP a few days after formation (both *in vitro* and *in vivo*) while there seem to be two distinct populations expressing or not Cathepsin K (121, 122). Miron et al. also discussed the polarization potential of MNGCs, in parallel with the polarization of macrophages, with a proposed distinction between pro-inflammatory M1-MNGCs that were also called foreign body giant cells (FBGCs) and wound-healing M2-MNGCs. It is impossible to state whether the suggested M2-MNGCs are the MNGCs observed in close contact to the CaP materials leading to bone formation or if M2-MNGCs can differentiate further into true osteoclasts even if this last statement seems unlikely due to their unresponsiveness to RANKL *in vitro* (54). In our hypothesis, M2-MNGCs are likely to be involved in late stages of chronic inflammation, leading to fibrous encapsulation. In any case, there is an urgent need to better characterize those MNGCs and to discover the cell communications involved in their formation.

Preliminary results showed that conditioned media from MSC culture could have a positive direct impact on osteoclastogenesis (123). This effect of MSCs could rely on enhanced secretion or membrane expression of RANKL. Activated T cells were also reported to increase osteoclastogenesis *in vitro* (124) but they cannot be the main source of RANKL in MSC-based bone formation as many successful experiments were carried out in Nude mice. Also, a number of factors are known to influence osteoclastogenesis, primarily by modifying RANKL/RANK signaling (125). *In vitro*, TGFβ (a known product of MSC but also Treg) promote osteoclast formation from RANKL stimulated precursors but also decreases RANKL expression in osteoblasts resulting in fewer osteoclasts in co-culture (126). In mice, activation of the non-canonical Wnt pathway by Wnt5a in osteoclast precursors increases the production of RANK (127). These are only few examples of molecules that could be implicated in the MSC-osteoclast communications and future studies will certainly better delineate this key step toward MSC-CaP induced bone formation.

As the newly formed bone comes mostly from host osteoblasts, it entails recruitment and differentiation of new MSCs or the newly characterized subset of skeletal stem cells [SSC, (128)]. We hypothesize that osteoclasts might be the essential attractor for those cells, setting off a local bone remodeling cycle. The basic mechanisms and the major signaling molecules involved in the osteoclast-osteoblast crosstalk during the physiological coupling of bone resorption and formation are well described (129, 130). Osteoclasts are known to release growth factors from the degradation of bone matrix and, most importantly in our case, to express chemotactic and osteogenic coupling factors toward cell of the osteoblastic lineage such as BMP6, WNT10b, and S1P (131). The CTHRC1 protein, expressed by mature osteoclasts, promote osteoblastic differentiation *in vitro* and an osteoclast-specific KO induce a low bone mass phenotype in mice (37). More recently, an important study unveiled a reverse signaling mechanism whereby osteoclasts secrete extracellular vesicles expressing RANK which are able to stimulate membrane RANKL on the surface of osteoblasts to induce bone formation (132). Also, as osteoclasts can degrade the biomaterial, they modulate the local calcium and phosphate concentrations, thus influencing the deposition of the apatite layer and the calcium sensing of other cell types (26, 133).

Simultaneously to this main phenomenon, MSCs are likely to induce a switch from M1 macrophages to the M2 phenotype, the formation of regulatory dendritic cells and the suppression of B, NK, CD4+, and CD8+ T cells while promoting Th2 and Treg cells. The timing of activation of the various cells is critical as the initial acute inflammation is necessary to recruit all the immune cells but is detrimental if it becomes chronic and favors the formation of MNGCs. The M1/M2 balance of macrophages phenotype has a key role in this switch to resolve inflammation and move on to bone formation (85, 134). Moreover, the M2 phenotype favored by MSCs is thought to help in late stages of osteoblastic differentiation and mineralization (86). The stressful conditions and, eventually, the apoptosis of implanted MSCs might increase their inherent immunomodulatory properties.

## Conclusion

**Figure 3:**
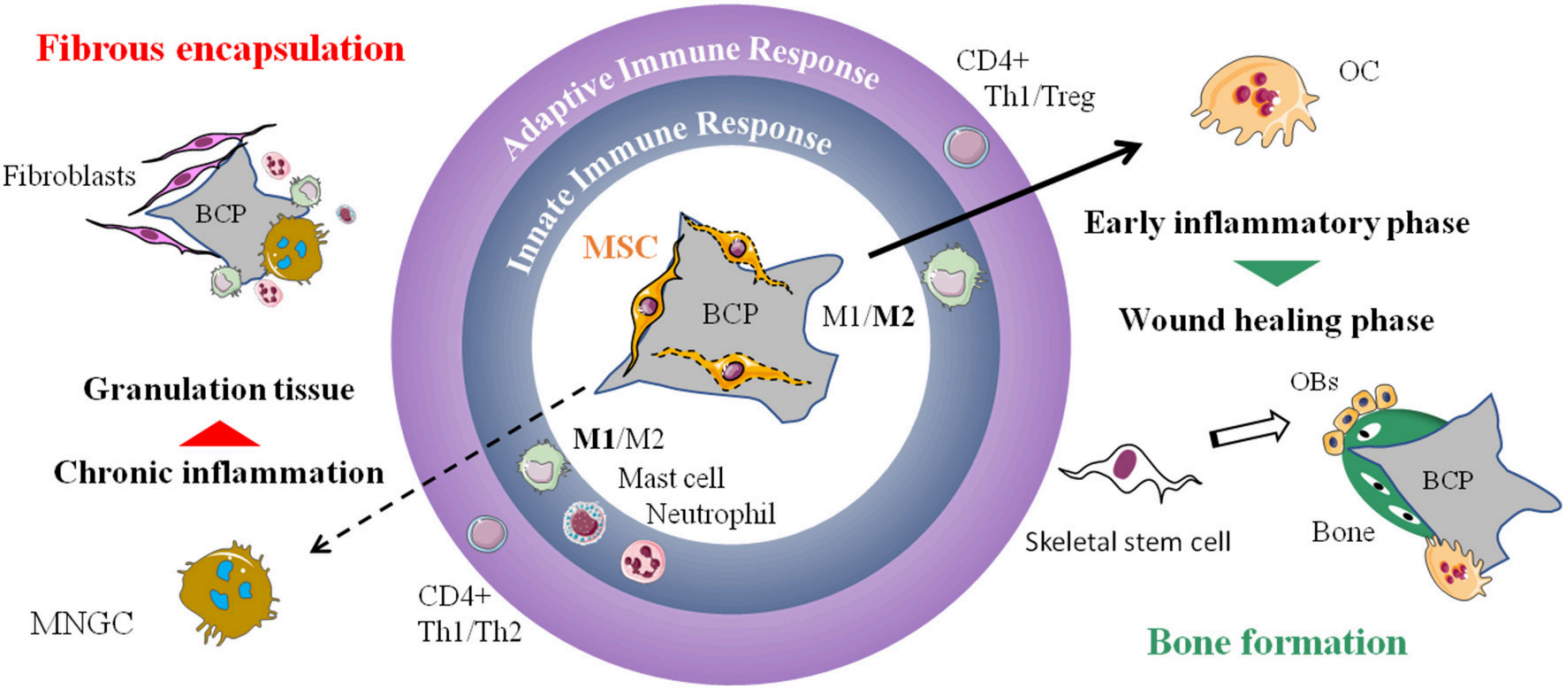
Proposed mechanism of MSC-CaP immune modulation leading to bone formation. The local innate and adaptive immune response will determine the fate of the implanted biomaterial (central part of the drawing). On the left, is displayed the classical foreign body reaction characterized by activation of M1 macrophages, mast cells, neutrophils, Th1, and Th2 CD4+ lymphocytes. It leads to the formation of MNGCs, chronic inflammation and subsequent fibrous encapsulation of the implant. On the right, adjunction of MSCs to the biomaterial favor M2 macrophages, Th1, Treg, and osteoclastogenesis followed by recruitment of new stem cells, likely from the skeletal subtype, that differentiate into bone forming osteoblasts. MSC, mesenchymal stem cell; BCP, biphasic calcium phosphate; M1, pro-inflammatory macrophages; M2, alternatively activated macrophages; Th1/Th2/Treg, type 1 helper/type 2 helper/regulatory T cells; MNGC, multi-nucleated giant cell; OC, osteoclast; OBs, osteoblasts.

The implantation of CaP biomaterials in combination with MSCs emphasizes the central role of the host immune system in bone regeneration. It is important to consider that the cellular events hypothesized here may only occur on an osteoconductive CaP material. The implanted MSCs potentiate the effect of the biomaterial allowing ectopic bone formation by creating a bone-like microenvironment. We highlighted here the pivotal role that macrophages and osteoclasts play in the multistep process of bone formation induced by MSC-CaP implantation (Figure 3) but this complex mechanism is just beginning to be explored. Over the course of several weeks, multiples cells types and molecules appear implicated in a coordinated manner before bone is formed. Any dysregulation would lead to unwanted chronic inflammation and fibrosis. A better comprehension of these spatiotemporal cell communications is mandatory to reach more efficient bone healing and develop better cell-free approaches.

## Author Contributions

All authors participated in the literature search, organization, writing, reviewing, and proofreading of the manuscript. PH, ND, VT, and FB designed the figures. MB and PL created the tables.

### Conflict of Interest Statement

ND is employed by Instructure Labs, B.V. The remaining authors declare that the research was conducted in the absence of any commercial or financial relationships that could be construed as a potential conflict of interest.
